# An Oral Formulation of the Probiotic, *Bacillus subtilis* HU58, Was Safe and Well Tolerated in a Pilot Study of Patients with Hepatic Encephalopathy

**DOI:** 10.1155/2020/1463108

**Published:** 2020-06-30

**Authors:** Sayed Yossef, Frances Clark, Sarah S. Bubeck, John Abernethy, Thomas Bayne, Kiran Krishnan, Aicacia Young

**Affiliations:** ^1^Sayed Yossef, Inc., 3304 Stones Throw Ave, Poland, OH 44514, USA; ^2^Bubeck Scientific, 194 Rainbow Dr. #9418, Livingston, TX 77399, USA; ^3^Amk Research, 1026 SW 2^nd^ Ave D, Gainesville, FL 32601, USA; ^4^Microbiome Labs, 101 E Town Place, Suite 210, Saint Augustine, FL 32092, USA

## Abstract

**Background:**

Hepatic encephalopathy often results in high blood ammonia levels because of inefficient ammonia processing by the liver. Lactulose treatment promotes the growth of urease-producing gut bacteria and a reduced colon pH, thus reducing blood ammonia absorption. It is thought that probiotics as an add-on therapy may be beneficial. *Patients and Methods*. *Bacillus subtilis* HU58 was tested for safety and tolerability in patients with hepatic encephalopathy taking lactulose in this double-bind, placebo-controlled, 4-week pilot study. Study participants received one dose of *B*. *subtilis* HU58 or placebo (orally) for the first five days and two daily doses thereafter. Participants were monitored for safety and blood ammonia levels.

**Results:**

Forty patients participated (placebo, 11; probiotic, 29). Baseline characteristics were generally comparable; the mean baseline blood ammonia level was somewhat higher in the probiotic group. Mild or moderate treatment-emergent adverse events (TEAEs) were reported in 27.3% and 17.2% of patients in the placebo and probiotic groups, respectively; no severe TEAEs were reported. One patient (9.1%) taking placebo and two (6.9%) taking the probiotic experienced serious TEAEs (SAEs); none resulted in study discontinuation and all were considered to have no/unlikely relationship to the study product. There were no significant differences in the mean percent change (MPC) of blood ammonia levels between groups, though the probiotic group exhibited a trend toward a milder increase. Stratification of the probiotic group by baseline blood ammonia level (>60 *μ*g/dL and ≤60 *μ*g/dL) resulted in a significantly reduced MPC in the >60 *μ*g/dL subgroup (MPC (SD); ≤60 *μ*g/dL (*n* = 14), 35.3% (73.3); >60 *μ*g/dL (*n* = 14), −26.5% (24.4); *p* = 0.0087).

**Conclusions:**

Daily treatment with oral *B*. *subtilis* HU58 was safe and well tolerated over a 4-week period in patients with hepatic encephalopathy, and a significantly reduced MPC of blood ammonia level was observed in patients with a baseline level >60 *µ*g/dL.

## 1. Introduction

Ammonia is a byproduct of normal gut protein digestion by bacteria and mucosal enzymes which is subsequently detoxified by the liver in healthy individuals [1]. Patients with hepatic encephalopathy experience accumulation of ammonia in their blood because of insufficient removal by the liver, often due to acute or chronic liver disease. This buildup of ammonia in the blood results in hyperammonemia [1].

Ammonia is neurotoxic; elevated levels in the blood may result in decreased excitatory neurotransmission [2] as well as brain swelling. Patients with elevated blood ammonia may have symptoms such as abnormal behavior and compromised cognition which can progress to more severe manifestations. The primary treatment for hepatic encephalopathy is the reduction of ammonia absorption into the blood from the intestinal lumen, often accomplished by treating the patient with lactulose or lactitol. These nonabsorbable disaccharides promote the growth of nonurease producing bacteria and acidification of the colon, both of which result in reduced ammonia absorption [3]. Patients with recurrent hepatic encephalopathy may be treated with rifaximin in addition to lactulose [1].

*Bacillus subtilis* is a Gram-positive, rod-shaped, spore-forming, aerobic/facultative anaerobic bacterium that is found in many environments, though the principle reservoir is soil [4]. More recently, it is recognized that some *B*. *subtilis* strains are commonly found in the intestinal tracts of animals [4–6] and that these bacteria should be considered gut commensals [7]. One such strain, *B*. *subtilis* HU58, was isolated from human feces. It is sporulation positive and was shown to sporulate, germinate, proliferate, and resporulate in the gastrointestinal tract of mice [5].

Probiotics are live, nonpathogenic microorganisms which, when given in adequate quantities, confer a health benefit to the host [8]. There is growing interest in the use of *Bacillus* spp. in probiotic formulations [9] because they have several characteristics that make them particularly suitable for probiotic use. These characteristics include a high level of resistance to desiccation and heat allowing for long-term storage at room temperature, as well as stability over a wide range of pHs, enabling survival as the bacterium travels through the low pH of the stomach [7]. *Bacillus* spp. have been used historically in a variety of fermented foods [10] which are thought to be associated with a range of health benefits. Multiple health benefits have been attributed to *Bacillus* probiotic use, though the mechanisms behind this are not well understood; clinical trials of health benefits reported for *Bacillus* probiotic strains are reviewed in Elshaghabee et al. [9]. Probiotics, such as *B*. *subtilis*, are expected to alter the gut microbiome and promote the growth of nonurease producing organisms, thus reducing ammonia production. Additionally, the presence of *B*. *subtilis* in the gut may further reduce ammonia, as these microorganisms can utilize ammonia. Such qualities indicate that *B*. *subtilis* may be useful for reducing ammonia levels in patients with hepatic encephalopathy. In fact, probiotics have been proven useful for prevention or amelioration of hepatic encephalopathy in patients with cirrhosis [11, 12] and there is current interest in using a pharmabiotic approach for treating hyperammonemia [13]; however, high-quality randomized clinical trials are needed to further explore this [14].

The purpose of this double-blind, placebo-controlled pilot study was to determine the safety and tolerability of a probiotic formulation of *B*. *subtilis* HU58 when used in combination with lactulose in patients with hepatic encephalopathy.

## 2. Methods

### 2.1. Patients

Patients ≥21 years of age with a history of elevated ammonia levels, who were currently taking a stable dose of lactulose and had stable ammonia levels were eligible for this study. Ammonia levels were considered stable if the patient had two readings in the same range consecutively in the 3 months prior to study enrollment. Lactulose dose was considered stable if there were no dose changes in the 3 months prior to study enrollment. Patients were excluded if they had participated in any clinical research study within the previous 8 weeks, were encephalopathic, or were allergic to either probiotic formulations or rice. All patients provided written informed consent.

### 2.2. Study Design and Treatments

**Figure 1:**
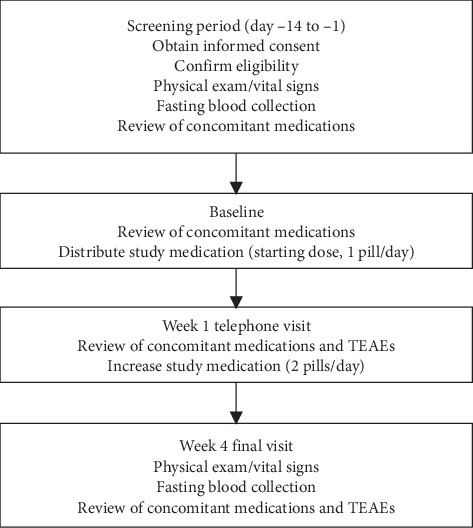
Study design. TEAE, treatment-emergent adverse event.

This manuscript describes a single-center, double-blind, placebo-controlled, 4-week pilot study to determine the safety and tolerability of a probiotic formulation of *B*. *subtilis* strain HU58 when used with lactulose. The study design is shown in Figure 1. Patients underwent three office visits (screening (day −14 to −1), baseline (day 1), and final visit (week 4)) and one phone visit (week 1). Eligible patients were screened prior to starting the study. The screening visit included a physical exam, blood collection, and a review of concomitant medications. Informed consent was also obtained at this visit. Both the baseline office visit and phone visit included a review of concomitant medications. The phone visit also included a review of treatment-related adverse events (TEAEs). The final office visit included a physical exam, blood collection, and a review of concomitant medications and TEAEs. All blood samples were collected under fasting conditions.

The study product provided 5 billion colony-forming units (CFUs) of *B*. *subtilis* HU58 per capsule (Microbiome Labs, Saint Augustine, FL, USA; manufactured in Scottsdale, AZ, USA) for oral dosing; the placebo was the same sized capsule filled with rice flour. The placebo and study product had a similar appearance and were dosed in the same way to maintain study blinding.

Starting from the baseline (day 1) visit, patients took one capsule daily (probiotic or placebo) for the first 5 days of the study; the dose was then increased to two capsules daily (probiotic or placebo) for the remainder of the study. The purpose of the dose escalation was to help ensure the patients did not experience TEAEs. Patients remained on lactulose as prescribed by their physician at the beginning of the study. The rationale for dose selection for this probiotic study product was based on results from consumer use and prior published studies [15, 16].

The use of concomitant medications, including over-the-counter medications, was prohibited during the study (with the exception of vitamins and contraceptives) unless deemed by the investigator as necessary to treat TEAEs. Any such medication use was recorded in the case report form.

Restrictions during the study included no change in lactulose dose; no probiotic supplements other than the study product; no excessive intake of probiotics from food; no grapefruit-containing foods or beverages; no herbal, mineral, or dietary supplements; no consumption of alcohol; and no smoking or use of nicotine. If a patient withdrew from the study, the reason for withdrawal was recorded and whenever possible, the patient underwent all assessments required at the final visit. Patients failing to return for the final assessment were contacted by clinical personnel in an attempt to achieve patient compliance. If a patient withdrew from the study, they were not replaced.

This study was conducted in accordance with the ethical principles of the Declaration of Helsinki and all applicable government regulation with respect to the International Conference on Harmonisation Good Clinical Practice Guideline. The study protocol and informed consent forms were approved by an institutional review board (IRB #201502695) at Advarra (formerly Schulman Associates Institutional Review Board, Inc.). The study was conducted at Sayed Yossef, MD, Inc. (Poland, OH).

### 2.3. Primary Objective

The primary objective of this study was to determine the safety and tolerability of a probiotic formulation with *B*. *subtilis* HU58 when used with lactulose. Safety and tolerability were assessed by monitoring for TEAEs and serious TEAEs (SAEs). This study was not powered for efficacy outcomes.

### 2.4. Secondary Objective

The secondary objective of this study was to determine any change in ammonia levels. Changes in ammonia levels were assessed by determining blood ammonia levels at the screening and final visits and comparing the percent change from baseline to the end of study between the two groups.

### 2.5. Statistical Methods

Sample size was not determined by a formal calculation; 40 patients were considered sufficient to achieve the study objectives for this pilot study. The safety analysis set (SAF) included all patients who received at least one dose of the study product. Descriptive statistics (number and percentage) were used to summarize TEAEs by severity and SAEs. Percent change was calculated to normalize the differences in the baseline blood ammonia levels. The difference in the mean of the percent change of blood ammonia levels from baseline between the probiotic and placebo groups was calculated using Welch's *t*-test to allow for unequal variance. A *p* value of <0.05 was considered significant. Statistical analyses were performed using GraphPad Prism 8 (GraphPad Software, La Jolla, CA).

## 3. Results

### 3.1. Patients

**Figure 2:**
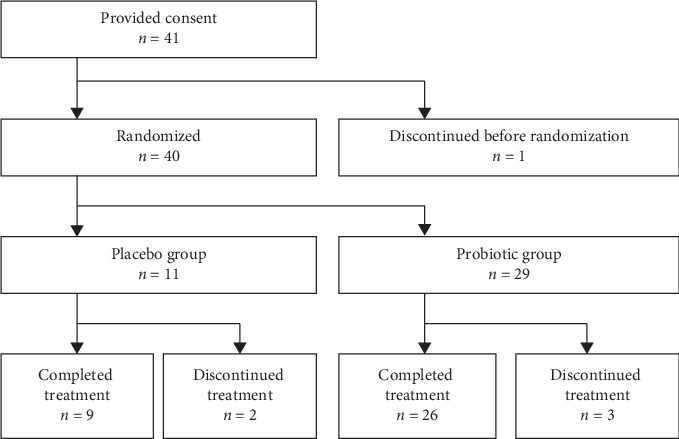
Patient disposition.

This study was conducted between October 25, 2015, and February 1, 2016. The disposition of patients is shown in Figure 2. Consent was obtained from 41 patients; one patient was lost to follow-up before randomization. Forty patients were randomized; 11 to the placebo group and 29 to the probiotic group. Of those, 9 and 26 patients in the placebo and probiotic groups, respectively, completed the study (Figure 2). Two patients in the placebo group discontinued before completion of the treatment period because of serious TEAEs (*n* = 1) and loss to follow-up (*n* = 1). Three patients in the probiotic group discontinued before completion of the study because of withdrawal of consent (*n* = 1) and loss to follow-up (*n* = 2). The SAF included 40 patients who received the study product or placebo; the patient who consented but was not randomized was not included in the analyses.

The baseline characteristics of the patients are shown in Table 1 and were generally comparable between the groups (sex, race/ethnicity, age, body mass index (BMI)). The mean (standard deviation (SD)) baseline blood ammonia levels were 54.4 (30.3) and 67.0 (44.7) *μ*g/dL for the placebo and probiotic group, respectively. Although the difference in ammonia levels between the two groups was not statistically significant (*p* = 0.1203), it was considered large (12.0 *μ*g/dL) and a higher proportion of patients in the probiotic group than in the placebo group had a baseline blood ammonia level >60 *μ*g/dL (50% and 20%, respectively).

### 3.2. Safety

A total of six TEAEs occurred in three patients in the placebo group and 10 occurred in five patients in the probiotic group.  Table 2 summarizes the incidence, severity, and relationship to the study treatment of the reported TEAEs. TEAEs were mild in two (18.2%) and three (10.3%) patients in the placebo and probiotic groups, respectively, and a respective one (9.1%) and two (6.9%) patients experienced moderate TEAEs. No patients in either group experienced severe TEAE. One SAE (hyperglycemia) was reported in one patient in the placebo group. Two patients in the probiotic group experienced SAEs. Of these, one patient experienced two SAEs (two occurrences of lower extremity pain and finger cramping) and one patient experienced one SAE (respiratory failure). None of the SAEs resulted in study discontinuation and all SAEs were considered to have no or an unlikely relationship to the study product.

One patient in the placebo group discontinued the study due to mild TEAEs (spurs of brightness and floaters/shadows in the left eye, slight headache). Of the 6 and 10 TEAEs reported in the placebo and probiotic groups, respectively, 3 (50.0%) and 1 (10.0%) were possibly related to the study treatment, and none were likely related to the study treatment. All other TEAEs were considered unrelated or unlikely related to the study treatment (Table 2). There were no deaths reported during the study.

### 3.3. Blood Ammonia Levels

**Figure 3:**
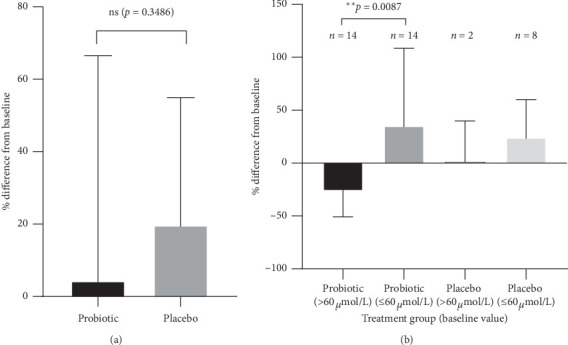
Changes in blood ammonia levels (a) by treatment group and (b) for patients stratified by baseline blood ammonia level; placebo group: *n* = 10, probiotic group: *n* = 28 (ammonia data were missing for 1 patient in each group); ns = not significant;^∗ ∗^*p* ≤ 0.01.

The difference in mean percent change (MPC) from baseline between the probiotic (MPC, 4.4%; SD, 62.14) and placebo (MPC, 19.8%; SD, 35.12) groups was 15.4%, which was not significant (*p* = 0.3486) (Figure 3(a)). Patients in the probiotic group demonstrated a trend for less of an increase in blood ammonia levels compared with the placebo group. Patients in the probiotic group were stratified according to their baseline blood ammonia level (>60 *μ*g/dL and ≤60 *μ*g/dL). Those with a baseline blood ammonia level >60 *μ*g/dL (*n* = 14) experienced a decrease (MPC, −26.5%; SD, 24.4) in blood ammonia levels over the course of the study, while those with a level ≤60 *μ*g/dL (*n* = 14) experienced an increase (MPC, 35.3%; SD, 73.3). This difference was statistically significant (*p* = 0.0087) (Figure 3(b)). It was not possible to perform statistical analysis of the difference between the placebo and probiotic groups in stratified patients because there were too few patients with a baseline blood ammonia level >60 *μ*g/dL in the placebo group (*n* = 2) (Figure 3(b)).

## 4. Discussion

Daily treatment with the oral probiotic, *B*. *subtilis* HU58 (5 billion CFUs/capsule; first 5 days on study, 1 capsule/day; study day 6 to end of study, 2 capsules/day) was safe and well tolerated in patients with hepatic encephalopathy taking lactulose. No major safety concerns were reported during this 4-week pilot study. No significant differences in MCP of blood ammonia levels between patients in the placebo and probiotic groups were observed. However, after stratifying the patients in the probiotic group by baseline blood ammonia level, we noted a significant difference in the MPC in those who had a baseline blood ammonia level >60 *μ*g/dL compared with ≤60 *μ*g/dL. Blood ammonia levels were decreased in the group with a baseline value >60 *μ*g/dL, suggesting that these patients may derive a greater benefit from probiotic treatment. Larger studies are needed to better understand the clinical significance of this finding.

The incidence of TEAEs was low (placebo, *n* = 3 (27.3%); probiotic, *n* = 5 (17.2%)); all were mild or moderate and though similar between the two treatment groups, the incidence was somewhat higher in the placebo group. Only one patient discontinued the study due to TEAEs; this patient was in the placebo group. Most TEAEs were considered unrelated or unlikely related to the study treatment (placebo, 50%; probiotic, 90%). Taken together, these findings indicate that TEAEs were not due to the probiotic.

The safety results are in line with previous reports of *B*. *subtilis* including reports that this strain (HU58) and others have been isolated from the gastrointestinal tracts of healthy individuals [4–6] and the previously reported use of this strain as part of a commercially available probiotic cocktail [17]. To our knowledge, this is the first study examining the safety of *B*. *subtilis* HU58 probiotic as add-on therapy in patients with hepatic encephalopathy being treated with lactulose.

The MPC of blood ammonia levels was positive in both patient populations (indicating an overall increase in levels at the end of study). However, there was a trend for a smaller increase for patients in the probiotic group compared with those in the placebo group. This difference was not significant, and it should be noted that there was quite a large SD in both groups. It is known that blood ammonia levels can vary widely [18] and future studies would benefit from larger patient groups.

The data were further analyzed by stratifying the patients in the probiotic group by baseline blood ammonia levels. There was a significant difference between the MPC for patients with >60 *μ*g/dL (−26.52%) compared with ≤60 *μ*g/dL (35.26%). The observed reduction in blood ammonia levels in the patients who had a higher baseline level is potentially of clinical importance. While larger studies of longer duration are needed to confirm these results, patients with a higher baseline blood ammonia level may experience a greater benefit from probiotic treatment. Patients with >60 *μ*g/dL in the placebo and probiotic groups could not be compared as there were only 2 patients meeting this criterion in the placebo group. It should be noted that a lower percentage of patients in the placebo group than in the probiotic group had baseline blood ammonia levels >60 *μ*g/dL (placebo, 20%; probiotic, 50%). Analysis based on baseline blood ammonia level stratification was not planned. Therefore it was not taken into consideration during randomization.

While the use of blood ammonia levels to diagnose and monitor hepatic encephalopathy is somewhat controversial [19], there are studies that support it [20, 21]. One such study performed a retrospective analysis of data from a randomized, double-blind study of patients with cirrhosis and showed a correlation between fasting levels of blood ammonia and the relative risk of hepatic encephalopathy episodes [21]. Another study reported that elevated arterial ammonia levels were associated with advanced hepatic encephalopathy and indicated a poor prognosis [22]. While using blood ammonia levels to diagnose and monitor hepatic encephalopathy remains controversial, it is accepted that these levels play an important role in the etiology of this disease, likely being a major factor in the development of hepatic encephalopathy [23].

Clinical trials using *B*. *subtilis* HU58 alone have not been previously reported in either healthy subjects or patients with hepatic encephalopathy. However, trials using other probiotics have been conducted in patients with liver disease. A prospective, randomized controlled trial in which patients with cirrhosis who had not yet developed hepatic encephalopathy were prophylactically treated with either placebo or probiotics (VSL#3, Sigma-Tau Pharmaceuticals) reported that probiotics were effective in preventing the development of hepatic encephalopathy and reducing blood ammonia levels after three months of daily treatment [24]. Additionally, a Cochrane review evaluated 21 reported studies that looked at the use of various probiotics at various doses in patients with hepatic encephalopathy [14]. Regarding blood ammonia levels, the authors concluded that while there was an overall trend for a reduction with the use of probiotics, well-planned studies are needed to provide high-quality data in support of probiotic use.

This study contributes information that will be useful to plan future studies to better evaluate the association between probiotic use, particularly *B*. *subtilis* HU58, and outcomes in patients with hepatic encephalopathy. Though preliminary, this study provides insight on clinical parameters that are potentially important to consider when designing such trials.

The findings of this pilot study are limited because of the small sample size and the short duration of the study. Future studies with larger study groups are warranted. It should be confirmed whether patients with baseline blood ammonia levels >60 *μ*g/dL experience a greater blood ammonia reduction from probiotic use than those with levels ≤60 *μ*g/dL and whether this reduction could contribute to an improved overall prognosis. The duration of probiotic use may also impact the results; therefore, studies in which patients are treated with probiotics for an extended time-period should be conducted. In addition, the use of multistrain probiotic formulations should be investigated, as they may provide additional benefits to these patients.

## 5. Conclusion

Once daily oral ingestion of 5–10 billion CFUs *B*. *subtilis* HU58 was safe and well tolerated in hepatic encephalopathy patients during this 4-week study. While the difference in MPC of blood ammonia levels between the placebo and probiotic groups was not significant, a trend toward a lower end of study MPC (i.e., closer to baseline) was observed in the probiotic group. When the patients in the probiotic group were stratified by baseline blood ammonia level, there was a significant difference in the MPC of patients with a baseline level of >60 *μ*g/dL compared with ≤60 *μ*g/dL. Additionally, this patient group experienced an overall reduction in blood ammonia levels at the end of the study. The findings of this pilot study provide important information for planning future, larger studies to evaluate the clinical benefits of *B*. *subtilis* HU58 probiotic either alone or in combination with other probiotic bacterial strains.

## Figures and Tables

**Table 1:** Baseline patient characteristics.

	Placebo, *N* = 11	Probiotic, *N* = 29
Sex^a,b^		
Male	5 (50)	16 (59)
Female	5 (50)	11 (41)
Race/ethnicity^a,b^		
Caucasian	10 (100)	25 (73)
African American	0 (0)	2 (7)
Age, years^a,b^	62.9 ± 9.2	65.6 ± 11.4
Body weight, kg^a,b^	98.3 ± 19.7	89.4 ± 25.2
BMI, kg/m^2a,b^	35.1 ± 9.0	32.6 ± 10.1
Ammonia level^c,d^, *μ*g/dL	54.4 ± 30.3	67.0 ± 44.7
≤60	39.6 ± 15.6 (*n* = 8)	36.4 ± 13.5 (*n* = 14)
>60	103.5 ± 6.4 (*n* = 2)	97.6 ± 44.1 (*n* = 14)

Values are presented as *n* (%) or mean ± standard deviation. ^a^Demographic data missing for 1 patient in the placebo group. ^b^Demographic data missing for 2 patients in the probiotic group. ^c^Ammonia data missing for 1 patient in the placebo group. ^d^Ammonia data missing for 1 patient in the probiotic group. BMI: body mass index.

**Table 2:** Incidence of treatment-emergent adverse events.

	Placebo (*N* = 11), *n* (%)	Probiotic (*N* = 29), *n* (%)
Patients with at any TEAE	3 (27.3)	5 (17.2)
Mild	2 (18.2)	3 (10.3)
Moderate	1 (9.1)	2 (6.9)
Severe	0	0
SAE	1 (9.1)	2 (6.9)
TEAEs leading to permanent discontinuation of the study product	1 (9.1)	0
Relationship of TEAE with study treatment		
Unrelated (/total TEAEs)	3/6 (50.0)	5/10 (50.0)
Unlikely related (/total TEAEs)	0	4/10 (40.0)
Possibly related (/total TEAEs)	3/6 (50.0)	1/10 (10.0)
Likely related (/total TEAEs)	0	0
Deaths	0	0

SAE: serious treatment-emergent adverse event; TEAE: treatment-emergent adverse event.

## Data Availability

All of the data used to support the findings of this study are included within the article.
